# Reply to Papageorgopoulou et al. The Aftermath of Bariatric Surgery: Can the Average Emergency Surgeon Deal with Its Complications? Comment on “Zawadzka et al. Current Knowledge and Perceptions of Bariatric Surgery among Diabetologists and Internists in Poland. *J. Clin. Med.* 2022, *11*, 2028”

**DOI:** 10.3390/jcm11123533

**Published:** 2022-06-20

**Authors:** Karolina Zawadzka, Krzysztof Więckowski, Tomasz Stefura, Piotr Major, Magdalena Szopa

**Affiliations:** 1 2nd Department of General Surgery, Faculty of Medicine, Jagiellonian University Medical College, 30-688 Krakow, Poland; karolina.zawadzka@onet.pl (K.Z.); k.wieckowski@student.uj.edu.pl (K.W.); tomasz.stefura@gmail.com (T.S.); piotr.major@uj.edu.pl (P.M.); 2 Centre for Research, Training and Innovation Jagiellonian (CERTAIN Surgery), 30-688 Krakow, Poland; 3 Department of Metabolic Diseases, Faculty of Medicine, Jagiellonian University Medical College, 30-688 Krakow, Poland

The World Health Organization (WHO) has identified obesity and overweight as an epidemic of the 21st century. As the number of obese people in the world increases, so does the amount of bariatric surgery performed. The most recent IFSO Worldwide Survey reported that 696,191 bariatric operations were performed worldwide in 2018 [1]. Therefore, a great need has arisen for surgeons, including those not performing bariatric surgery on a daily basis, to be familiar with the pathophysiological effects of these operations and to know the management of early and late complications that may occur after bariatric surgery.

We carefully read the comment by Papageorgopoulou et al. [2]. We appreciate raising interesting issues related to the subject of our article [3]. They show the surgeon’s perspective and suggest that similar education on bariatric surgery, as discussed in our article, should apply to general surgeons. According to the literature, up to 15% to 30% of patients will visit the emergency room or require admission within 3 years after bariatric surgery. Interestingly, nearly three-quarters of the emergency surgeons expressed concern when they were asked to treat the acute abdomen in those patients who had undergone bariatric surgery [4]. Therefore, we strongly agree with Papageorgopoulou et al., that there is a need to train all surgeons in bariatric surgery, especially to adequately treat emergencies resulting from complications after this type of surgery. It would certainly be useful to intensify the training in bariatrics, especially as part of any specialization in surgery.
